# Detecting Protein Communities in Native Cell Extracts by Machine Learning: A Structural Biologist’s Perspective

**DOI:** 10.3389/fmolb.2021.660542

**Published:** 2021-04-15

**Authors:** Fotis L. Kyrilis, Jaydeep Belapure, Panagiotis L. Kastritis

**Affiliations:** ^1^Interdisciplinary Research Center HALOmem, Charles Tanford Protein Center, Martin Luther University Halle-Wittenberg, Halle (Saale), Germany; ^2^Institute of Biochemistry and Biotechnology, Martin Luther University Halle-Wittenberg, Halle (Saale), Germany; ^3^Biozentrum, Martin Luther University Halle-Wittenberg, Halle (Saale), Germany

**Keywords:** cellular homogenates, random forest, convolutional neural network, cryo-EM, mass spectrometry, structural biology, protein–protein interactions, metabolons

## Abstract

Native cell extracts hold great promise for understanding the molecular structure of ordered biological systems at high resolution. This is because higher-order biomolecular interactions, dubbed as protein communities, may be retained in their (near-)native state, in contrast to extensively purifying or artificially overexpressing the proteins of interest. The distinct machine-learning approaches are applied to discover protein–protein interactions within cell extracts, reconstruct dedicated biological networks, and report on protein community members from various organisms. Their validation is also important, e.g., by the cross-linking mass spectrometry or cell biology methods. In addition, the cell extracts are amenable to structural analysis by cryo-electron microscopy (cryo-EM), but due to their inherent complexity, sorting structural signatures of protein communities derived by cryo-EM comprises a formidable task. The application of image-processing workflows inspired by machine-learning techniques would provide improvements in distinguishing structural signatures, correlating proteomic and network data to structural signatures and subsequently reconstructed cryo-EM maps, and, ultimately, characterizing unidentified protein communities at high resolution. In this review article, we summarize recent literature in detecting protein communities from native cell extracts and identify the remaining challenges and opportunities. We argue that the progress in, and the integration of, machine learning, cryo-EM, and complementary structural proteomics approaches would provide the basis for a multi-scale molecular description of protein communities within native cell extracts.

## Introduction

Since the dawn of biological research, humans are breaking-apart living systems to understand their structure and function. For example, in *Book VI of History of Animals*, Aristotle systematically addressed the processes of egg formation and chick embryo development by visual inspection. Nowadays, with the rapid technological advances in biochemical, biophysical, structural, and computational methods, cellular homogenates can be understood in great detail, providing network and structural information of the biomolecules within them. Crude extracts made by the lysis of cellular material possess operative aspects of cellular function, but in a context that is easier to manipulate. They are biotechnologically exploited for bioproduction (Karim and Jewett, 2016), cell-free gene expression, transcription, translation (Silverman et al., 2020), and, recently, molecular design (Hammerling et al., 2020). Probing the intrinsic structure of cell extracts is of paramount importance, so that their function is understood in detail. Until recently, the study of cell extracts was limited to low-resolution data (Han et al., 2009), but, with methodological advances, the resolution of 4.7 Å for the biomolecular complexes within those was reached (Kastritis et al., 2017).

Recent studies not only increased the achievable resolution (Arimura et al., 2020; Ho et al., 2020; Su et al., 2021), particularly in the membrane (Su et al., 2021) or nuclear extracts (Arimura et al., 2020) but also determined the snapshots of higher-order organization of in-extract flexible, functional metabolons (Kyrilis et al., 2021). The importance and challenges of integrative structural studies of native extracts and the correlation between structural disorder and function for in-extract metabolons were recently reviewed (Kyrilis et al., 2019; McCafferty et al., 2020; Skalidis et al., 2020). Reaching the milestone of near-atomic detail a few years ago proved that native cell extracts are amenable to structural studies and considerably broadened the structural proteomics field by expanding the concept of “protein communities” (Kastritis et al., 2017), primarily described by Gavin et al. (2006). Protein communities describe the associated molecules of several macromolecular complexes arranged in close proximity encoding functionally synchronized biomolecular entities. For example, they may efficiently transfer substrates along with enzymatic pathways [dubbed *metabolons*, reviewed in (Kastritis and Gavin, 2018)], effectively transduce signals, and regulate protein synthesis on local cellular demand. However, their inherent complexity limits probing their intrinsic structure to a few abundant biomolecular complexes, e.g., functional pyruvate dehydrogenase higher-order architecture (Kyrilis et al., 2021). The review of machine-learning approaches that are already applied in various intermediate analysis steps demonstrates an optimistic perspective in addressing this issue, and thus allowing a deeper understanding of protein communities in the future. In this study, by machine learning, we refer to the un-/supervised algorithms that are trained to learn the patterns in the scientific data retrieved from -omics, cryo-electron microscopy (cryo-EM), or any other method to predict the desired physically meaningful feature without human intervention.

## Higher-Order Complexity of Protein Communities: An Ideal Test Bed for Machine Learning

Protein communities (or, in general, biomolecular communities) are endogenously present in the cell and can be retrieved in native cell extracts. They are composed of biomolecular assemblies of varying compositional and chemical heterogeneity. A protein community comprises a functional cellular assembly and encodes localized functions (e.g., as in the case of metabolons). Protein communities also include interconnected protein complexes in variable stoichiometry and, therefore, represent a holistic view of cellular function beyond the description of their individual constituents. Due to their intricacy, communities must be characterized with an array of methods: (a) -omics methods, especially quantitative mass spectrometry (MS), to identify constituent molecules; (b) activity assays to probe their function; (c) cross-linking to find the interacting community biomolecules; (d) large-scale molecular modeling or cryo-EM characterization of community members to annotate complexes within the protein communities; and (e) cryo-EM characterization to visualize protein communities. This multi-scale, integrative characterization of protein communities can only be performed in native cell extracts and was previously discussed (Kyrilis et al., 2019). This integrative, systematic analysis was performed for eukaryotic communities involved in the synthesis of fatty acids (Kastritis et al., 2017) and in the metabolism of oxoacids (Kyrilis et al., 2021).

In this review, we outline the methods and challenges faced in such integrative studies of protein communities. Furthermore, we assess and discuss the state-of-the-art machine-learning methods applied in adjoint problems that could better aid investigations in this field. In the first two sections, we discuss the molecular characterization of protein communities, first in crude and then in simplified lysates. The next two sections describe the structural characterization of protein community members, since structural analysis of complete protein communities is a formidable task. This is because cryo-EM of complete protein communities can show ultrastructural features, but does not provide high-resolution three-dimensional (3D) reconstructions due to the highly complex and intricate structure of the community. We finally surveyed published machine-learning tools that are principally developed for diverse characterization of the biomolecular complexes. In each subsequent section, we discuss the applicability, promises, and limitations of machine-learning methods for deciphering protein communities.

## Predicting Protein Communities in Crude Native Cell Extracts

**FIGURE 1:**
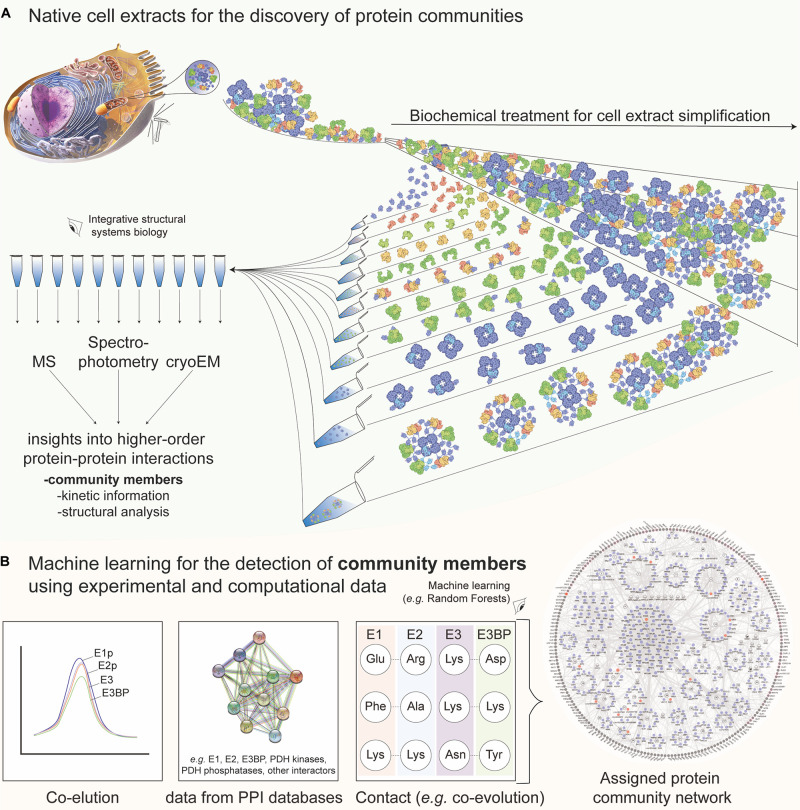
Native cell extracts as a tool for discovering protein communities with the aid of machine learning. **(A)** Methods to experimentally extract identity, structure, and dynamics information of protein communities. In short, the cell is lysed and the subsequent fractionation is applied to recover co-eluting protein material. In a large-scale manner, mass spectrometric, kinetic, and cryo-EM analysis of the fractions leads to the characterization of protein communities in native cell extracts. The example of the pyruvate dehydrogenase complex (PDHc) metabolon is shown. Molecular representations for PDHc are retrieved and further edited from Protein Data Bank “Molecule of the Month” section [Source: Image from the RCSB PDB September 2012 Molecule of the Month feature by David S. Goodsell (10.2210/rcsb_pdb/mom_2012_9)]. The cell representation on the top left was retrieved from Microsoft PowerPoint 2019 v16.47. **(B)** Combined data regarding protein–protein interactions stemming from fractionation (co-elution), external database information (network data), and contact information prediction (e.g., from co-evolution analysis, chemical cross-linking or mutagenesis experiments) among community members are used for machine learning, e.g., using a random forest. Finally, a network with interconnected protein communities is derived and insights into community members can be retrieved. External data shown are extracted from STRING (https://string-db.org/) and network shown from Kastritis et al. (2017). E1, E2, E3, and E3BP are the proteins structuring the 10-MDa complex of the PDHc metabolon, all involved in the complex reaction of pyruvate oxidation.

Cell extracts are amenable to biochemical treatment to probe the biomolecular content (Figure 1A), and methods were applied to study the retrieved homogenate directly (i.e., breaking the cellular material and subjecting it to an array of characterization tools). Proteins present in the cell extracts can be studied by MS, providing identification for thousands of protein sequences (Beck et al., 2011; Titeca et al., 2019). Unfortunately, this information offers a list of proteins, and, optimally, a report on their relative abundance, but not on their interactions. To predict communities, network analysis must then be performed by integrating the external interaction data for community members or their close homologs as, e.g., initially performed for the interconnected yeast complexes using tandem affinity purification (TAP) and MS (Gavin et al., 2002). In recent studies, experimental and/or computational methods for characterizing protein–protein interactions (PPI) are included, connecting *in vivo*, *in vitro*, and *in silico* data (Rao et al., 2014). By meticulous data integration, considering the strengths and limitations of each approach that was applied to discover PPIs (Rao et al., 2014), a network is then constructed using the machine-learning (Havugimana et al., 2017) method. In particular, interesting computational approaches for PPI prediction include, but are not limited to, a combination of different machine-learning models to take a majority vote for final prediction (Saha et al., 2014), a game theory-based approach inspired by a non-cooperative sequential game (Maulik et al., 2017), and deep neural networks that either incorporate physical/chemical properties and graph theory (Zhang and Kabuka, 2019) or combine with decision-tree classifiers for the final PPI prediction (Wang et al., 2019).

Naturally, training sets are of vital importance for reconstructing a biological network and are mostly extracted from the PPI databases such as CORUM (Giurgiu et al., 2019), IntAct (Hermjakob et al., 2004), and GO (Harris et al., 2004). The availability of a high-confidence set of PPIs is often limited, especially when it comes to organisms that lack genome, transcriptome, and/or proteome data. Even in well-studied organisms, the construction of a *confusion matrix* (*error matrix*) for PPIs is not an easy task. Proteins dynamically interact, change localization, and can even alter their function due to moonlighting (Jeffery, 2014), and therefore, according to the cellular state and environmental conditions, PPIs may differ. Such discoveries revealed localized variations in interaction networks of disease phenotypes (Vidal et al., 2011), and, recently, severe acute respiratory syndrome coronavirus 2 (SARS-CoV-2) cellular interactors (Gordon et al., 2020). Protein networks are, therefore, commonly employed in biotechnological and medical applications because the cellular function is probed in a holistic approach, complementing mechanistic investigations into molecular recognition. Traditionally, reconstruction of protein networks is not only essential for characterizing protein complexes, but also for their higher-order interactions present in their communities (Gavin et al., 2002, 2006).

## Simplifying Protein Community Detection Within Cell Extracts by Integrating Co-Elution Data and Chemical Cross-Linking

The increased complexity of cellular homogenates brings various limitations in the study of their biomolecular content, mainly because of the well-known bias toward the identification of high-abundant proteins and complexes (Fursch et al., 2020). An idea to confidently annotate proteins in cell extracts, retrieve more interactors, and optimize the robust identification of protein communities is to subject the extracted homogenate to a subsequent biochemical treatment that would coarsely separate the biomolecular complexes on a certain biophysical property (termed protein co-fractionation, e.g., using the hydrodynamic radius as performed *via* size-exclusion chromatography (SEC) of the native cell extract). Mapping fractionated extracts with various proteomics methods was recently reviewed (Salas et al., 2020). The application of co-fractionation to monitor protein associations (Havugimana et al., 2012; Kristensen et al., 2012) perhaps stems from previous works that measured the enzymatic activities across retrieved cellular fractions, e.g., in the fractionated extracts of *Escherichia coli*, where interactions of Krebs cycle enzymes were probed (Barnes and Weitzman, 1986). Nowadays, the high-resolution separation of cell extracts is mostly performed by using high-resolution SEC coupled to MS (Salas et al., 2020). This method (a) simplifies the cell extract according to an intrinsic physical property of the contained biomolecules; (b) provides per-fraction quantitative data regarding protein abundance and co-detection; and (c) offers robust per-protein elution profiles across the studied fractions, which may be used for subsequent integration into a PPI network. Protein co-fractionation can be used to identify interactors within protein communities (Kastritis et al., 2017) and compare PPI networks across species, highlighting evolutionary implications (Wan et al., 2015). An example of data integration to derive a PPI network, highlighting protein communities, is shown in Figure 1B.

As with the previously described PPI networks, the application of machine-learning approaches is crucial, not only to integrate the protein co-elution data but also to discriminate random co-elution events from true (interacting) protein complexes. The machine-learning-based tools to probe the complexes within cell extracts of different organisms were developed (Kastritis et al., 2017; Stacey et al., 2017; Hu et al., 2019; Fossati et al., 2020). EPIC (Hu et al., 2019), an open-source software tool, may specifically use co-elution data to predict protein complexes found in cell extracts after training and validating a random forest algorithm (Tin Kam, 1995) or a support vector machine algorithm (Boser et al., 1992). The random forest algorithm showed superior performance when applied to predict co-eluting complexes and their communities after cross-validation from *Caenorhabditis elegans* (Hu et al., 2019), *Chaetomium thermophilum* (Kastritis et al., 2017), and HeLa cells (Fossati et al., 2020). Recently, PCprophet incorporated Bayesian inference to identify altered protein profiles across experiments that probe phenotypic changes (Fossati et al., 2020). Predicting protein communities from co-fractionation data rely on complex inference from the resulting network after reconstructing it with identified PPIs. Due to the density of the network, partitioning methods to recover protein complexes are limited, and often graph clustering algorithms that handle weighted graphs to generate overlapping clusters are applied [e.g., ClusterONE (Nepusz et al., 2012), or the more recent, ONCQS (Zhao and Lei, 2019)]. High-density chemical cross-linking can, therefore, offer complementary data to enrich and validate true protein co-elution and protein complex/community member data (Sinz, 2018). Cross-linking was applied to soluble extracts (Liu et al., 2015; Gotze et al., 2019), membrane complexes (Larance et al., 2016), large macromolecular complexes to dissect conformational flexibility (Tuting et al., 2020), and, importantly, directly within the SEC fractions where proteins are determined to co-elute for the characterization of protein communities (Kastritis et al., 2017). Algorithms to detect co-eluting PPIs (Elias and Gygi, 2007; Havugimana et al., 2012) or cross-links (Ji et al., 2016; Huang et al., 2020) can include machine-learning tools to probe the complexity of high data dimensionality.

## Processing (Cryo-)Em Images From Native Extracts With a Focus on Machine Learning

Using cryo-EM imaging of native cell extracts to structurally analyze protein communities is essential. This is because proteomics methods discover the sequences of the community members or their interactions but do not provide information on their higher-order structure within their communities. Even if high-density cross-linking retrieves interacting proteins and their relative interacting distances, the community structure is unknown, including stoichiometry. It is noted that deriving stoichiometry for protein communities is not trivial, and a combination of cryo-EM, immunoblotting data, MS, and cross-linking MS in fractionated extracts was recently performed to derive approximate stoichiometry for the higher-order structure of the endogenous pyruvate dehydrogenase complex (Kyrilis et al., 2021). Direct methods, such as electron microscopy, can, therefore, be applied to observe cell extracts and were previously used in combination with MS at low resolution to visualize protein complexes (Han et al., 2009). However, recently, with advances in cryo-EM (Kuhlbrandt, 2014), native cell extracts delivered high-resolution data (Kastritis et al., 2017) and the first images of protein communities involving fatty acid synthase (FAS) together with other megadalton complexes (Kastritis et al., 2017). Recent results in the field also showed that abundant complexes can be reconstructed *de novo* (Ho et al., 2020), but not as members of protein communities. We also recently communicated the structural and functional characterization of communities involved in oxo acid metabolism by integrative methods (Kyrilis et al., 2021). Despite these advances, the high complexity of the imaged cell extract hinders proper quantification and 3D reconstruction of the interacting molecules within the extracts, and this is because of multiple issues regarding the specimen complexity. Therefore, most of the algorithms that were developed are applied to protein complexes and not to their higher-order assemblies in their native communities.

**FIGURE 2:**
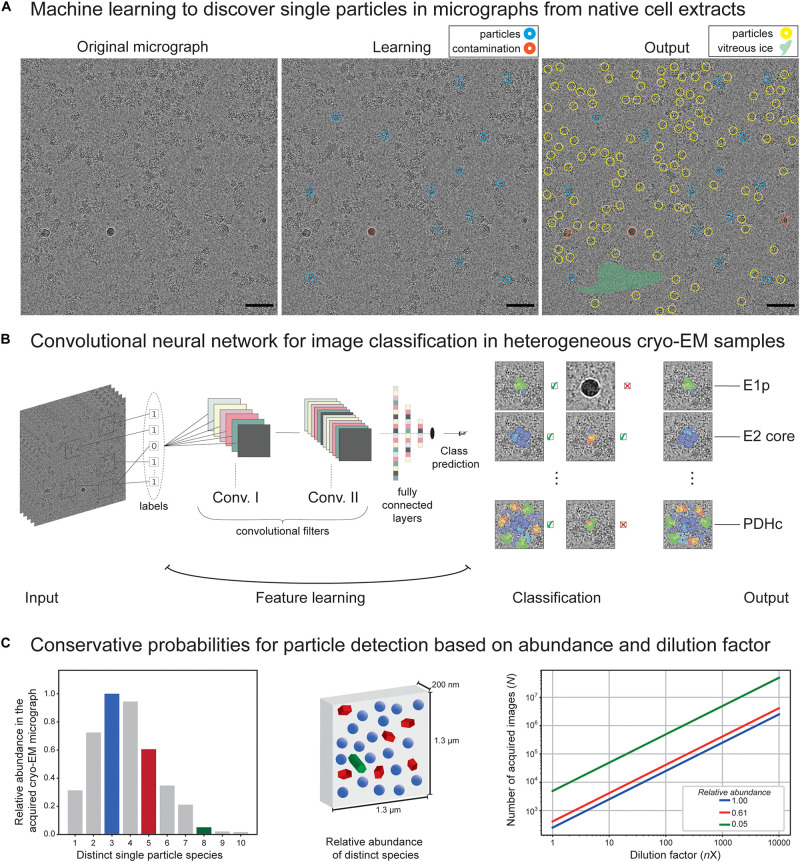
Application of machine learning on cryo-EM images derived from native cell extracts. **(A)** A cryo-electron micrograph from *C. thermophilum* fractionated cell extracts is shown. During machine learning, the algorithm is being trained to discriminate particles from contamination, vitreous ice, aggregation, and noise. At the end, the algorithm optimally picks and selects learned features that were not previously recognized during learning. Red circles indicate contamination, and blue and yellow circles indicate learned and predicted particles. Size of the circle does not match particle size but represents a correctly picked particle. Green highlighted area signifies empty regions of vitreous ice recognized by the algorithm. **(B)** Structure of a convolutional neural network algorithm frequently used to detect signal in cryo-EM micrographs. Input micrographs are used for feature learning during the convolution step of algorithm training. Optimal training would lead to efficient classification of the single particles and/or their higher-order assemblies and discriminate those from noise, contamination, and aggregates. A final output is achieved with metabolon members in their unbound and bound states as recognized by the convolutional neural networks in heterogeneous cryo-EM micrographs of native cell extracts. **(C)** Conservative probabilities for particle detection based on abundance and dilution factor. In the left panel, an example of 10 distinct single-particle species is shown with their relative abundance following an assumed T-squared distribution. In the middle panel, an illustration of relative particle abundance for three distinct particles (blue, green, and red, representing high, medium, and low abundant species in a calculated 4K × 4K micrograph with a pixel size of 3.17 Å and thickness of 200 nm) is shown. In the right panel, dependency of the number of images required to reach ∼5,000 single particles on the dilution factor is shown (assuming no biochemical manipulation for particle enrichment).

Cryo-EM micrographs contain two-dimensional (2D) projections of the particles in different orientations but are inherently of low contrast and often include contamination or undesirable features (see, e.g., Figure 2A). The signal-to-noise ratio in typical cryo-EM tomographs is ∼0.1, perhaps comparable to imaging in astronomy. Except in cryo-EM, multiple short exposures are recorded. The traditional methods, such as bandpass, or Wiener filtering (Jain and Seung, 2008; Sindelar and Grigorieff, 2011; Xie et al., 2012), to improve the contrast are insensitive to the underlying noise properties. The cryo-EM field recently witnessed a surge in machine-learning models that are trained to learn the noise characteristics and offer better denoising [(Bepler et al., 2020) and references. therein].

The traditional template-based approaches [e.g., (Huang and Penczek, 2004)] pick particle candidates by estimating the similarity of an image region to a reference, also known as a template, through cross-correlation techniques. The template-matching methods are prone to introduce template-based bias and are known for a high rate of false positives. This stems from the fact that, if matching is performed over enough number of random regions (e.g., noise only), then meaningless noise can be perceived as a pattern, a phenomenon dubbed as “Einstein-from-noise” (Shatsky et al., 2009). For the purpose of selecting desirable regions without a reference, deep learning algorithms were developed (Wang et al., 2016; Zhu et al., 2017; Punjani et al., 2017; Bepler et al., 2018; Tegunov and Cramer, 2019; Wagner et al., 2019; Zhang et al., 2019; Sanchez-Garcia et al., 2020b). Inspired by computer vision applications, using convolutional neural networks (CNNs) (Tegunov and Cramer, 2019; Sanchez-Garcia et al., 2020b), per pixel-image segmentation of particle/non-particle regions was demonstrated (Figure 2B). Many of these architectures are explicitly designed to eliminate undesirable features or implicitly learn to avoid them (Wang et al., 2016; Zhu et al., 2017; Bepler et al., 2018; Wagner et al., 2019; Zhang et al., 2019). Recent machine-learning and deep learning-based methods demonstrated improved accuracy and low false-positive rates (Wang et al., 2016; Punjani et al., 2017; Zhu et al., 2017; Bepler et al., 2018; Tegunov and Cramer, 2019; Wagner et al., 2019; Zhang et al., 2019; Sanchez-Garcia et al., 2020b). Since templates can be essentially seen as filters, CNNs are the most successful models for the task of image classification and particle picking, as they are trained to learn thousands of 2D filters (Rawat and Wang, 2017). We speculate that these algorithms if trained in the heterogeneous mixtures of cell extracts instead of single-particle datasets, are expected to effectively detect particles of varying shapes and sizes and separate them from the artifacts in the micrographs of cellular extracts to systematically retrieve members of protein communities. However, the learning algorithm would still need to address the subsequent challenging step of segregating and clustering the particles into correctly assigned classes and yet incorporate rotational as well as contrast transfer function (CTF) invariance. Another important aspect is how multiple distinct 3D reconstructions stemming from heterogeneous 2D projections can be achieved. This can be generally performed by the conventional cryo-EM classification methods, but here we refer to a more specific challenge of faithfully representing the true variability in the data sufficiently well to be used for protein community discovery. This is in contrast to current classification methods that only aim to homogenize the data subset to yield the highest possible resolution. This notion in the data analysis would eventually lead to average densities of the particles that may or may not participate in the same communities. Recently, Verbeke et al. (2020) applied the projection-slice theorem principles to group the particles into consistent subsets prior to 3D classification and, therefore, avoid guessing the number of underlying 3D shapes present in the data. Still, current methods, during the reconstruction of cryo-EM data, assume that sample heterogeneity originates from a small number of independent, distinct states; however, in reality, the number of distinct states is (often) unknown. This issue becomes more important when other specimens of increased complexity are considered. A method that addresses this issue by approximating the continuous 3D density function of a single particle is CryoDRGN (Zhong et al., 2021), a deep neural network-based algorithm. Recent machine-learning methods may improve the protein density of experimental cryo-EM maps, while the use of generative adversarial networks (GANs) trained on pairs of 3D atomic models and their noise-free cryo-EM maps is shown to generate a more realistic ground-truth 3D density map (Sanchez-Garcia et al., 2020a). An excellent discussion by the Scheres laboratory covers these aspects through the implementation of neural networks for simulated cryo-EM 3D reconstructions (Kimanius et al., 2021). Finally, for post-processing of cryo-EM maps, new machine-learning algorithms were developed to account for resolution anisotropy (Ramirez-Aportela et al., 2019; Sanchez-Garcia et al., 2020a).

For machine-learning models to work in the context of data stemming from cryo-EM micrographs of native cell extracts, it is reasonable to assume that they may efficiently be trained to pick and sort the community members by their heterogeneity. However, to construct the corresponding *de novo* 3D cryo-EM maps, novel *ab initio* algorithms should be developed to tackle this complexity. Moreover, the proximity calculations by accounting the Cartesian coordinates of the derived single particles in the cryo-EM micrographs can aid in understanding the protein complex interconnectivity within communities. It would further aid the detection and structural analysis of protein communities and their members.

## Model Building in Cryo-Em Maps From Native Cell Extracts Combined With Structure Prediction

Traditionally, protein complexes from the high-resolution cryo-EM reconstructions can be built because the purified constructs are used. Such approaches are well-established for cryo-EM, but, again, become a challenge for native cell extracts, where the identity of the reconstructed protein complexes and their interactors can be unknown. It is even more difficult to reconstruct such complexes when they are participating in higher-order assemblies, and therefore additional heterogeneity is manifested. cryo-EM may be used to visualize protein communities but, without complementary data, it cannot characterize their structure at a reasonable resolution. It is extremely challenging to determine the 3D models of isolated flexible complexes, but not their native interactions within protein communities. cryo-EM is unlikely to provide discovery or evidence of protein communities by itself without correlating the image information to proteomic, literature, and other sources of data. Interestingly, abundant, rigid complexes within communities can be retrieved at sub-nanometer resolution from native cell extracts, as in the cases of FAS (Kastritis et al., 2017) and pyruvate dehydrogenase complex (PDHc) (Kyrilis et al., 2021).

If high resolution is achieved for a given protein complex, and side-chain resolution is realistic, then multiple methods can be used to model the density, including, for example, cryoID (Ho et al., 2020), that may perform *de novo* model building, assuming that the proteome of the organism is available. However, if the resolution is more than ∼4.0 Å, then side-chain resolution is unattainable, and modeling methods must be ultimately employed [e.g., (Russel et al., 2012; van Zundert et al., 2016)]. In this case, only orthogonal identification methods may be applied to recover the map identity. This information can then be used for subsequent model building. To resolve this unknown density, the previously mentioned proteomic methods for network construction and community detection are of vital importance. Prior to the protein modeling methods, fold recognition should be the primary consideration for structural analysis and implementation of fast-fold search algorithm into the cryo-EM map is important, as proposed by Saha and Morais (2012). Of course, if complexes include other, non-protein components, the identification is laborious. For such scenarios, neural networks are developed to localize nucleotides as well (Mostosi et al., 2020), but machine learning should be expected to resolve cryo-EM densities stemming from multiple types of biological (macro-) molecules. To localize different chemical molecules in a cryo-EM map, a ground truth is required, i.e., the training set as pairs of cryo-EM maps and coordinates of chemical molecules in it. The hydrogen bonding patterns could then be recovered by calculating the geometrical properties of the modeled biomolecule(s) which are used to correlate chemical structure with portions of the cryo-EM maps and, ultimately, serve as input for machine learning.

The abundance of protein complexes within sequential fractions may be correlated to the corresponding structural signatures that were recovered by negative staining or cryo-EM, and therefore assign an identity to recovered structural signatures, which are also members of their respective communities (Kastritis et al., 2017). This was previously performed for *C. thermophilum* complexes using simple cross-correlation functions (Kastritis et al., 2017) but was limited to assigning abundant species. Theoretically, if the abundance of distinct single particles is expected to follow a T-squared distribution (Figure 2C, left panel) within a particular thick micrograph (1,300 nm × 1,300 nm × 200 nm, pixel size of 3.17 Å), then their relative abundance can be estimated (Figure 2C, middle panel). Without cell lysis (e.g., by cryo-electron tomography of a cell), a surprisingly high number of tilt series is required for less abundant particles to reach ∼5,000 single particles [e.g., enough for efficiently retrieving structural signatures of FAS (Kastritis et al., 2017) or PDHc (Kyrilis et al., 2021)]. After cell lysis and without biochemical enrichment, this effect further magnifies due to dilution (Figure 2C). It is important to note that, using cell extracts, protein complexes can be selectively biochemically enriched, and their conservative estimates are shown in Figure 2C. Nevertheless, rare species will be difficult to capture, and an extremely high amount of data will be required. In addition, capturing rare species will be algorithmically challenging. Therefore, we expect only abundant complexes to be captured and the abundant community members to be structurally characterized [as in the case of communities involved in oxo acid metabolism (Kyrilis et al., 2021)]. The availability of data for heterogeneous mixtures is still highly scarce. A possible bottleneck is the availability of both MS data and negative staining/cryo-EM data for sequential cellular fractions, preferentially from the same experiment because alterations in the organism biology can drastically alter recovered profiles. Another idea is to generate all possible protein folds from the sequences identified in the fraction using automated 3D structure prediction algorithms and, then, systematically fit those 3D models in the reconstructed densities. Such work has not been performed to date, mainly because current methods are limited to the study of a few abundant protein complexes present in the fractions (Kastritis et al., 2017; Verbeke et al., 2018; Arimura et al., 2020; Ho et al., 2020; Kyrilis et al., 2021; Su et al., 2021) and, sometimes, their communities (Kastritis et al., 2017; Kyrilis et al., 2021).

Protein structure prediction, in particular, recently witnessed advances, not only in traditional structure prediction methods [e.g., ROSETTA (Leman et al., 2020), I-TASSER (Roy et al., 2010)], but also in methods that are based on machine/deep learning (Torrisi et al., 2020), such as basic feed-forward neural network, CNN, recurrent neural network (RRN), and generative adversarial networks (GAN) (Torrisi et al., 2020). A recent example that excelled in the Critical Assessment of protein Structure Prediction [CASP, (Moult et al., 1995)], which is a blind protein structure prediction experiment, is AlphaFold2 developed by DeepMind. AlphaFold2 is based on an attention-based neural network system (Jumper et al., 2020) and was trained on all publicly available experimental 3D structures in the Protein Data Bank (PDB). Even if a fold can be recognized [and, currently thousands of those were predicted *via* machine-learning-based ROSETTA functions (Yang et al., 2020) and added in Pfam (Mistry et al., 2021)], it is still far from explaining the higher-order interactions captured within the cryo-EM map. For understanding the molecular recognition, large protein complex assembly and community function are still out of reach: only methods that include experimental data to drive the modeling process with physics-based potentials [e.g., HADDOCK (van Zundert et al., 2016), IMP (Russel et al., 2012)] can provide physically realistic models. It is noted that the Critical Assessment of PRotein–protein Interactions (CAPRI) (Janin et al., 2003) is a blind experiment where algorithms are tested in their ability to solve the biomolecular recognition problem. To date, in CAPRI, the top-performing algorithms are physics-based which integrate experimental data from various targets.

## Discussion: Aspiring Deeper Structural Characterization of Protein Communities

Machine/deep learning is applied to a multitude of optimization problems that are related with the recovery and characterization of protein communities at high resolution. In each step toward their multi-scale molecular characterization, distinct approaches are applied, fitted to answer diverse questions arising from experimentally measured multidimensional data. Unambiguous and large training sets, avoiding overfitting and careful cross-validation, true test sets, and, overall, systematic benchmarking are all required to accurately predict the desirable outcome. However, the complex nature of native cell extracts has not yet been fully explored systematically from a structural perspective, especially in (a) deriving 3D reconstructions out of the cryo-EM data in an un-/supervised manner, (b) model building in the recovered 3D maps, and (c) interconnecting multi-scale structural information from (a) and (b) to discover structural data about protein communities. As of note, cryo-electron tomography of complex specimen and associated image processing methods for in-tomogram particle detection and classification (Xu et al., 2011, 2019; Chen et al., 2013; Zhou et al., 2020) may also inspire methods for chemically heterogeneous single-particle datasets (and vice versa) for future applications in the characterization of protein communities. Structural biology of native cell extracts, therefore, provides an ideal test bed for the development and application of artificial intelligence. It is of paramount importance to note that the studies of native cell extracts and the structural characterization of protein communities that reside within should not simply focus on retrieving high resolution. The extreme flexibility and heterogeneity of the participating biomolecules pose a practical limitation on the resolution; even if high resolution is achieved, it will be non-uniform and will be prohibitive for a deeper understanding of function. Instead, the studies should aim to characterize components, stoichiometry, and, *via* cryo-EM, to utilize structural data in the discovery of PPIs within communities. We expect that, in the years to come, more datasets for heterogeneous specimen will be available through dedicated databases [e.g., UNIPROT (UniProt Consortium, 2019), PRIDE (Perez-Riverol et al., 2019), CORUM (Giurgiu et al., 2019), EMDB (Lawson et al., 2011), EMPIAR (Iudin et al., 2016), and PDB (Berman et al., 2000)]. Given the exponential increase of open-source data, and significant advancement in computational hardware over the past decade, machine/deep learning algorithms will become more efficient. The machine-learning methods will be eventually able to tackle some of the aforementioned limitations in the analysis of complex mixtures and homogenates of soluble and/or membrane extracts with success, aiming to provide answers to the, yet, elusive conundrum of macromolecular recognition: *How and why biomolecules interact?*

## Data Availability Statement

The raw data supporting the conclusions of this article will be made available by the authors, without undue reservation.

## Author Contributions

PK conceived the review and wrote the manuscript with the contributions from FK and JB. FK made figures and collected the data presented in Figure 2A. The authors thank all eight reviewers for their highly valuable feedback and the subsequent conceptual improvements that were manifested in our manuscript. All authors contributed to the article and approved the submitted version.

## Conflict of Interest

The authors declare that the research was conducted in the absence of any commercial or financial relationships that could be construed as a potential conflict of interest.
